# Supplementary material for: End Sequence Analysis Toolkit (ESAT) expands the extractable information from single-cell RNA-seq data
Source: Genome Res. 2016 Oct;26(10):1397–410. doi: 10.1101/gr.207902.116 (PMC5052061; doi:10.1101/gr.207902.116)
Supplement: Supplemental Material [file supp_gr.207902.116_Supp_Materials.tar.gz › ESAT-esat_v0.1/doc/html/classbroad_1_1pda_1_1gene_1_1functional_annotation-members.html]

ESAT: Member List


ESAT


- Main Page
- Related Pages
- Packages
- Classes
- Files

- Class List
- Class Index
- Class Hierarchy
- Class Members

All Classes Namespaces Files Functions Variables Enumerator Pages

- broad
- pda
- gene
- functionalAnnotation

broad.pda.gene.functionalAnnotation Member List

This is the complete list of members for broad.pda.gene.functionalAnnotation, including all inherited members.

|  |  |  |
| --- | --- | --- |
| calcAllTermsEnrichment(Collection< Gene > geneSet, LinkedList< Collection< Gene >> randomGeneSets) | broad.pda.gene.functionalAnnotation |  |
| calcTermEnrichment(String term, HashMap< String, String > geneSet, LinkedList< HashMap< String, String >> randomSets, Double[] permPval, Double[] fischerExact, Integer[] numOverlap, LinkedList< Double > randomFischerExact, LinkedList< Integer > randomOverlap) | broad.pda.gene.functionalAnnotation |  |
| functionalAnnotation() | broad.pda.gene.functionalAnnotation |  |
| getTermGenes(String goTerm, BEDFileParser ref) | broad.pda.gene.functionalAnnotation |  |
| loadGoMap(String gofile, String goformat, int goSizeThreshold) | broad.pda.gene.functionalAnnotation |  |
| main(String[] args) | broad.pda.gene.functionalAnnotation | static |
| parseGoRawFiles(String Ontology, String Annotation, String pid2gid, String nm2pidF, int nmCol, int pidCol, String outf) | broad.pda.gene.functionalAnnotation | static |
| writeTermsEnrichments(String filename, int sizeThreshold, String str, ArrayList< String > termsSorted) | broad.pda.gene.functionalAnnotation |  |


---

Generated on Fri Aug 15 2014 15:10:59 for ESAT by  

 1.8.7
